# Engineering spatiotemporal patterns: information encoding, processing, and controllability in oscillator ensembles

**DOI:** 10.1088/2057-1976/ace0c9

**Published:** 2023-07-03

**Authors:** Walter Bomela, Bharat Singhal, Jr-Shin Li

**Affiliations:** 1 Department of Electrical and Systems Engineering, Washington University in St. Louis, United States of America; 2 Division of Biology & and Biomedical Sciences, Washington University in St. Louis, United States of America

**Keywords:** phase models, optimal tracking control, controllability, nonlinear oscillators

## Abstract

The ability to finely manipulate spatiotemporal patterns displayed in neuronal populations is critical for understanding and influencing brain functions, sleep cycles, and neurological pathologies. However, such control tasks are challenged not only by the immense scale but also by the lack of real-time state measurements of neurons in the population, which deteriorates the control performance. In this paper, we formulate the control of dynamic structures in an ensemble of neuron oscillators as a tracking problem and propose a principled control technique for designing optimal stimuli that produce desired spatiotemporal patterns in a network of interacting neurons without requiring feedback information. We further reveal an interesting presentation of information encoding and processing in a neuron ensemble in terms of its controllability property. The performance of the presented technique in creating complex spatiotemporal spiking patterns is demonstrated on neural populations described by mathematically ideal and biophysical models, including the Kuramoto and Hodgkin-Huxley models, as well as real-time experiments on Wein bridge oscillators.

## 1. Introduction

Effective regulation of synchronization patterns formed by a neural population is of practical importance to the treatment of neurological pathologies that often result from disruptions in these synchronization patterns. For example, motor dysfunctions in Parkinson’s disease are linked to excessive neural synchronization [1, 2], sleep disorders are caused by disturbances in the synchronized circadian rhythms [3], and disruption of clustered synchronous states in the brain is associated with cognitive dysfunctions [4]. Controlling neuron populations, possibly with a relatively small number of electrodes, is also critical for retinal implants, where the activation of a large number of sensory neurons in parallel and, more importantly, in the correct sequence, is required to transmit the visual information to the visual cortex [5].

Processing or encoding information using a network of neurons requires controlling the timing of neuronal spikes, which is described by complex mathematical models that make analysis and control design insurmountable. The complexity of the description of neural spiking can be significantly reduced by focusing only on the phase of the spiking dynamics, described using phase models [6]. These phase reduction approaches provide a direct link between the biophysical and computational models and weakly coupled phase oscillator models, enabling several insights into the nature of synchronous activity at the neuronal level, and have become a staple tool in computational neuroscience [7]. As a result of the effectiveness and simplicity of phase models, we utilize these simplified models for control design and analysis.

Robust and optimal control of either a single neuron or a population of neurons has been the subject of active research. This led to the development of novel control techniques for asymptotic and phase-selective entrainment of neural oscillators with periodic waveforms [8–12]; modulation of the spiking rates of neurons using charge-balanced and minimum-power controls [13, 14]; synchronization of neural populations [15, 16]; and desynchronization of globally coupled neurons [17–21]. Instead of regulating the whole population of neurons, selective spiking of neurons is also achieved by designing stimuli that induce firing of a subset of the population while inhibiting the rest [22]. On the other hand, the problem of suppressing hypersynchronous oscillations of the whole population during seizures is considered in [23].

The primary contribution of our work is to transform the challenging task of controlling dynamic structures in an ensemble of nonlinear oscillators into a tracking problem and to present a computationally tractable iterative algorithm to solve the formulated tracking problem. This tracking formulation does not require real-time feedback and is able to produce any desired phase configuration while simultaneously offering flexibility to accommodate the trade-off between different objectives, such as tracking errors and input energy. Moreover, we uncover the relationship between the information encoding capacity of a neuronal network and its controllability property and establish the conditions under which a network loses its encoding capacity. Specifically, we show that arbitrary phase patterns can be created by using a common control input (a stimulus) without requiring any specific coupling structure in a controllable neuron oscillator network, while only limited types of patterns are possible for a partially controllable network, which in turn reduces its information encoding capacity.

## 2. Methods

### 2.1. Phase reduction

The dynamics of a nonlinear oscillator, such as the Hodgkin-Huxley (HH) neuron model [24] or the limit cycle model for circadian rhythms [25], are often described by a set of coupled ordinary differential equations (ODEs) exhibiting a stable limit cycle. Consider a generic time-invariant dynamical model of an oscillating system, given by

\begin{eqnarray*}\dot{{\boldsymbol{x}}}={\boldsymbol{f}}({\boldsymbol{x}},u),\quad {\boldsymbol{x}}(0)={{\boldsymbol{x}}}_{0},\end{eqnarray*}

where ${\boldsymbol{x}}(t)\in {{\mathbb{R}}}^{n}$ is the state vector and $u(t)\in {\mathbb{R}}$ is the stimulus input. A system exhibiting an attractive, non-constant limit cycle ***γ*** (*t*) = ***γ*** (*t* + *T*), satisfying $\dot{{\boldsymbol{\gamma }}}(t)={\boldsymbol{f}}({\boldsymbol{\gamma }},0)$, on the periodic orbit ${\boldsymbol{\Gamma }}=\{{\boldsymbol{y}}\in {{\mathbb{R}}}^{n}:{\boldsymbol{y}}={\boldsymbol{\gamma }}(t)\ \mathrm{for}\ 0\leqslant t\leqslant T\}\subset {{\mathbb{R}}}^{n}$, can be reduced to a one-dimensional system, described by a nonlinear phase equation [11, 26–28] given by

\begin{eqnarray*}\dot{\theta }=f(\theta )+z(\theta )u(t),\end{eqnarray*}

where *θ* ∈ [0, 2*π*) is the phase variable, *f* and *z* are real-valued functions, and $u\in { \mathcal U }\subset {\mathbb{R}}$ is the external control [29–31] taken from the set of admissible control functions ${ \mathcal U }$. The function *f*, also referred to as the instantaneous frequency, represents the baseline dynamics of the oscillator in the absence of the control *u*, and *z* describes the response of the phase to *u* applied at a given phase *θ* and is referred to as the phase response curve (PRC) [32, 33].

### 2.2. Dynamic control of phase patterns

We formulate the phase pattern formation problem as an optimal tracking problem. Such a formulation offers the advantage of controlling the path to the desired phase pattern while simultaneously ensuring the optimality of the designed control input. Specifically, we consider a system involving a population of *n*-coupled neurons, as described by the phase model

**Figure 1.**
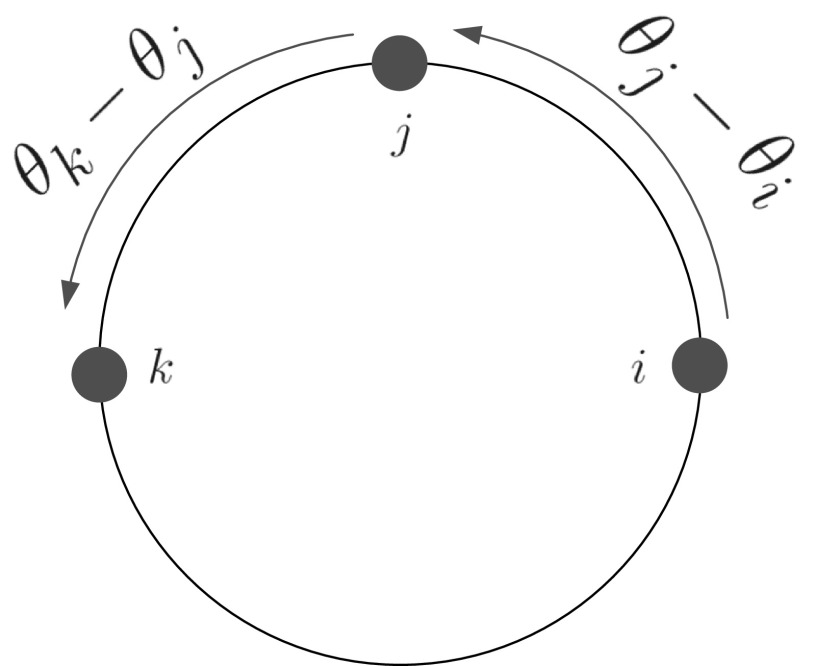
Description of phase assignment framework. With oscillator *i* as reference, one assigns the phases of oscillators *j* and *k* by imposing the desired phase differences *θ*_*j*_ − *θ*_*i*_ and *θ*_*k*_ − *θ*_*j*_, respectively.

\begin{eqnarray*}\dot{{\boldsymbol{\Theta }}}(t)={\boldsymbol{f}}({\boldsymbol{\Theta }})+{\boldsymbol{Z}}({\boldsymbol{\Theta }})u(t),\end{eqnarray*}

where ${\boldsymbol{\Theta }}(t)=({\theta }_{1},\cdots ,{\theta }_{n})^{\prime} \in [{\left.\mathrm{0,2}\pi \right)}^{n}\subset {{\mathbb{R}}}^{n}$, ${\boldsymbol{f}},{\boldsymbol{Z}}:[{\left.\mathrm{0,2}\pi \right)}^{n}\mapsto {{\mathbb{R}}}^{n}$, and $u(t)\in {\mathbb{R}}$ is the common control input broadcast to the ensemble. The drift ***f*** (Θ) = (*f*_1_(Θ), ⋯ ,*f*_*n*_(Θ)) contains the self- and coupling-dynamics of each oscillator in the network and ***Z*** (Θ) = (*z*_1_(*θ*_1_), ⋯ ,*z*_*n*_(*θ*_*n*_)) with *z*_*i*_(*θ*_*i*_) being the PRC of oscillator *i* for *i* = 1,…,*n*. The objective is to find an input stimulus *u*(*t*) that uses minimum energy to drive the system to follow a desired reference trajectory ${{\boldsymbol{\Theta }}}_{d}(t)=({\theta }_{{d}_{1}},\cdots ,{\theta }_{{d}_{n}})^{\prime} $. To this end, we first construct a desired reference **Θ**_*d*_(*t*) that results in the desired phase pattern (figure 1). After that, a control input that minimizes a quadratic cost functional, a combination of control energy and tracking error ***e*** (*t*) = **Θ**_*d*_(*t*) − **Θ**(*t*), is obtained by solving the dynamic optimization problem,

\begin{eqnarray*}\begin{array}{rcl}\mathop{\min }\limits_{u\in { \mathcal U }}\quad J &amp; = &amp; \varphi ({t}_{f},{\boldsymbol{e}}({t}_{f}))+{\int }_{0}^{{t}_{f}}{ \mathcal L }({\boldsymbol{e}}(t),u(t)){dt},\\ {\mathrm{s}}.{\mathrm{t}}.\dot{{\boldsymbol{\Theta }}}(t) &amp; = &amp; {\boldsymbol{f}}({\boldsymbol{\Theta }})+{\boldsymbol{Z}}({\boldsymbol{\Theta }})u(t),\\ {\boldsymbol{\Theta }}(0) &amp; = &amp; {{\boldsymbol{\Theta }}}_{0},\quad {\boldsymbol{\Theta }}({t}_{f})\ {\mathrm{is}}\,{\mathrm{free,}}\end{array}\end{eqnarray*}

where $\varphi :{\mathbb{R}}\times {{\mathbb{R}}}^{n}\mapsto {\mathbb{R}}$ and ${ \mathcal L }:{{\mathbb{R}}}^{n}\times {\mathbb{R}}\mapsto {\mathbb{R}}$ denote the terminal and the running cost, respectively. These costs depend on the formation error and are defined by $\varphi ({t}_{f},{\boldsymbol{e}}({t}_{f}))=\tfrac{1}{2}{\boldsymbol{e}}^{\prime} ({t}_{f})F{\boldsymbol{e}}({t}_{f})$ and ${ \mathcal L }({\boldsymbol{e}}(t),u(t))=\tfrac{1}{2}{\boldsymbol{e}}^{\prime} (t)Q(t){\boldsymbol{e}}(t)+R(t){u}^{2}(t)$, respectively, where *F* ≽ 0 and *Q*(*t*) ≽ 0 are *n* × *n* positive semi-definite matrices, and $R(t)\in {\mathbb{R}}$ is positive definite for all *t* ∈ [0, *t*_*f*_].

The optimal tracking problem, equation (4), can be solved using the principle of optimal control theory by considering its associated value function, *V*(*t*, *θ*). We derive the following solution to the minimization problem (4) (refer to the Supplementary Materials for the detailed analysis):

\begin{eqnarray*}{u}^{* }(t)=-{R}^{-1}{\boldsymbol{Z}}^{\prime} ({{\boldsymbol{\Theta }}}^{* })\left[P(t){{\boldsymbol{\Theta }}}^{* }(t)-{\boldsymbol{g}}(t)\right],\end{eqnarray*}

where the optimal trajectory Θ^*^(*t*) is the trajectory obtained by driving the system using the control input in equation (5) as follows

\begin{eqnarray*}{\dot{{\boldsymbol{\Theta }}}}^{* }(t)={\boldsymbol{f}}({{\boldsymbol{\Theta }}}^{* })+Z({{\boldsymbol{\Theta }}}^{* }){u}^{* }(t),\end{eqnarray*}

and $P(t)\in {{\mathbb{R}}}^{n\times n},g(t)\in {{\mathbb{R}}}^{n}$ satisfy a set of coupled differential equations given by

\begin{eqnarray*}\dot{P}(t)=P(t)E({\boldsymbol{\Theta }})P(t)-Q(t),\end{eqnarray*}

\begin{eqnarray*}\dot{{\boldsymbol{g}}}(t)=P(t)E({\boldsymbol{\Theta }}){\boldsymbol{g}}(t)+P(t){\boldsymbol{f}}({\boldsymbol{\Theta }})-Q(t){{\boldsymbol{\Theta }}}_{d}(t),\end{eqnarray*}

with the respective boundary conditions *P*(*t*_*f*_) = *F*, ***g*** (*t*_*f*_) = *F* **Θ**_*d*_(*t*_*f*_), and $E({\boldsymbol{\Theta }})={\boldsymbol{Z}}({\boldsymbol{\Theta }}){R}^{-1}(t){\boldsymbol{Z}}^{\prime} ({\boldsymbol{\Theta }})$.

The set of differential equations, in equations (5), (6), (7), and (8), characterizes the optimal control solution and is in general analytically and numerically intractable. To effectively solve this set of differential equations, a computational algorithm is also developed (algorithm 1). The developed iterative algorithm is computationally tractable and finds convergent tracking controls for the desired phase pattern if the ensemble system in (3) is controllable.

Algorithm 1. Algorithmic description of the iterative method.

**Table:** 

**Data**: ${\boldsymbol{f}}({\boldsymbol{\Theta }})$, ${\boldsymbol{Z}}({\boldsymbol{\Theta }})$, *Q*(*t*), *R*(*t*), *F*, ${\boldsymbol{\Theta }}({t}_{0})$, ${{\boldsymbol{\Theta }}}_{d}(t)$, *t*_*f*_
Initialization ;
*k* = 0;
${{\boldsymbol{\Theta }}}_{0}(t)={{\boldsymbol{\Theta }}}_{d}(t)$, ${{\boldsymbol{\Theta }}}_{0}(0)={{\boldsymbol{\Theta }}}_{d}(0)$;
**while** $| | {{\boldsymbol{\Theta }}}_{k}(t)-{{\boldsymbol{\Theta }}}_{k-1}(t)| | \gt \epsilon $ **do**
compute *P*_*k*_(*t*) and ${{\boldsymbol{g}}}_{k}(t)$ using ${\boldsymbol{Z}}({{\boldsymbol{\Theta }}}_{k}(t))$;
${u}_{k}(t)=-{R}^{-1}{\boldsymbol{Z}}^{\prime} ({{\boldsymbol{\Theta }}}_{k}(t))[{P}_{k}(t){{\boldsymbol{\Theta }}}_{k+1}(t)-{{\boldsymbol{g}}}_{k}(t)]$;
${\dot{{\boldsymbol{\Theta }}}}_{k+1}(t)={\boldsymbol{f}}({{\boldsymbol{\Theta }}}_{k+1}(t))+{\boldsymbol{Z}}({{\boldsymbol{\Theta }}}_{k}(t)){u}_{k}(t)$;
$k=k+1$;
**end**

### 2.3. Simulation and experimental details

We apply the proposed method to control the phase pattern formation in a network of HH neurons and Kuramoto oscillators [24, 29] (refer to the Supplementary Materials for the models and parameters used for simulations). The HH neuron model describes the generation and propagation of action potentials in a squid giant axon based on the dynamic interplay between ionic conductances and electrical activity [24], and serves as a canonical example for neural ensemble dynamics. Kuramoto oscillators are also frequently used to study the interaction of cortical brain areas [7, 34].

**Figure 2.**
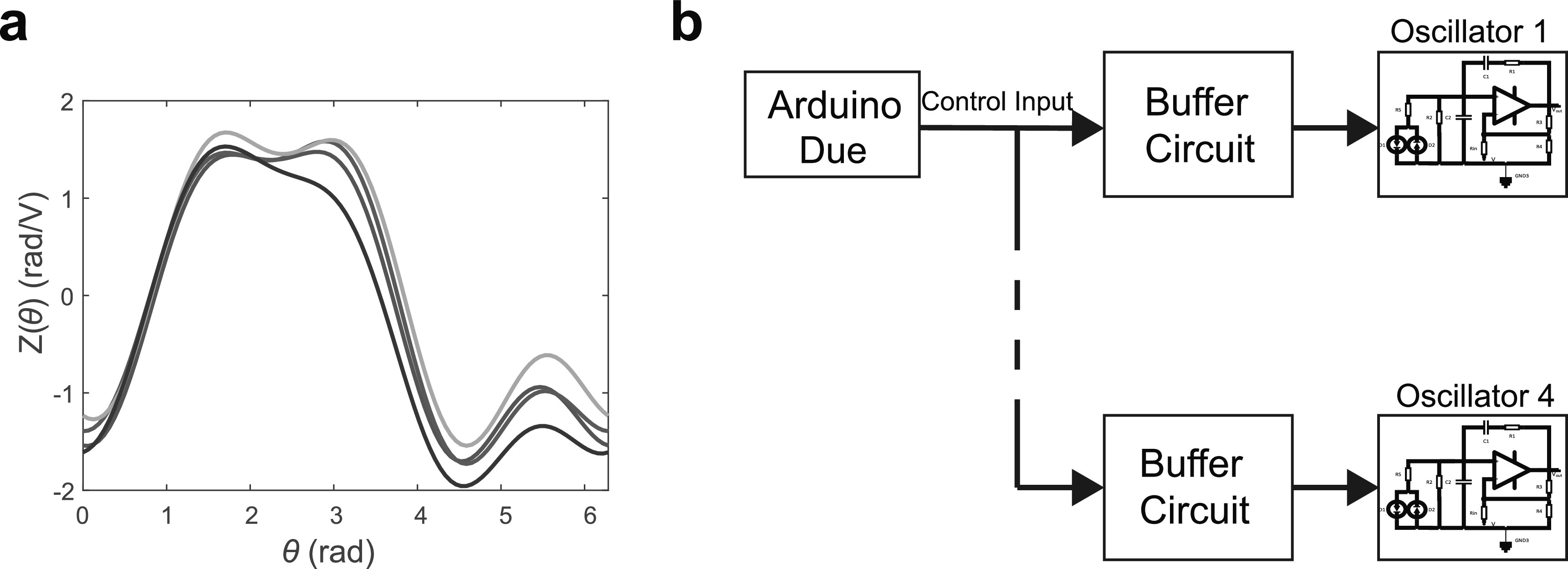
Real-time implementation of the control algorithm on Wien bridge oscillators. (a) Experimentally measured PRCs of the four oscillators. (b) Schematic diagram illustrating the real-time implementation setup.

To demonstrate the applicability of our method in real-world experimental settings and robustness against measurement noise, we test our method on a network of four Wien bridge oscillators. The experimental set-up consisted of four Wien bridge oscillators, buffer circuits connected to the input and output of each oscillator, and an Arduino Due microcontroller that measured the output voltages and was also used to apply the control input to the oscillators. The experimental setup along with the measured PRC of each oscillator is depicted in figure 2. The PRCs of the oscillators were experimentally measured by applying brief pulses and observing the phase shift of each oscillator.

**Figure 3.**
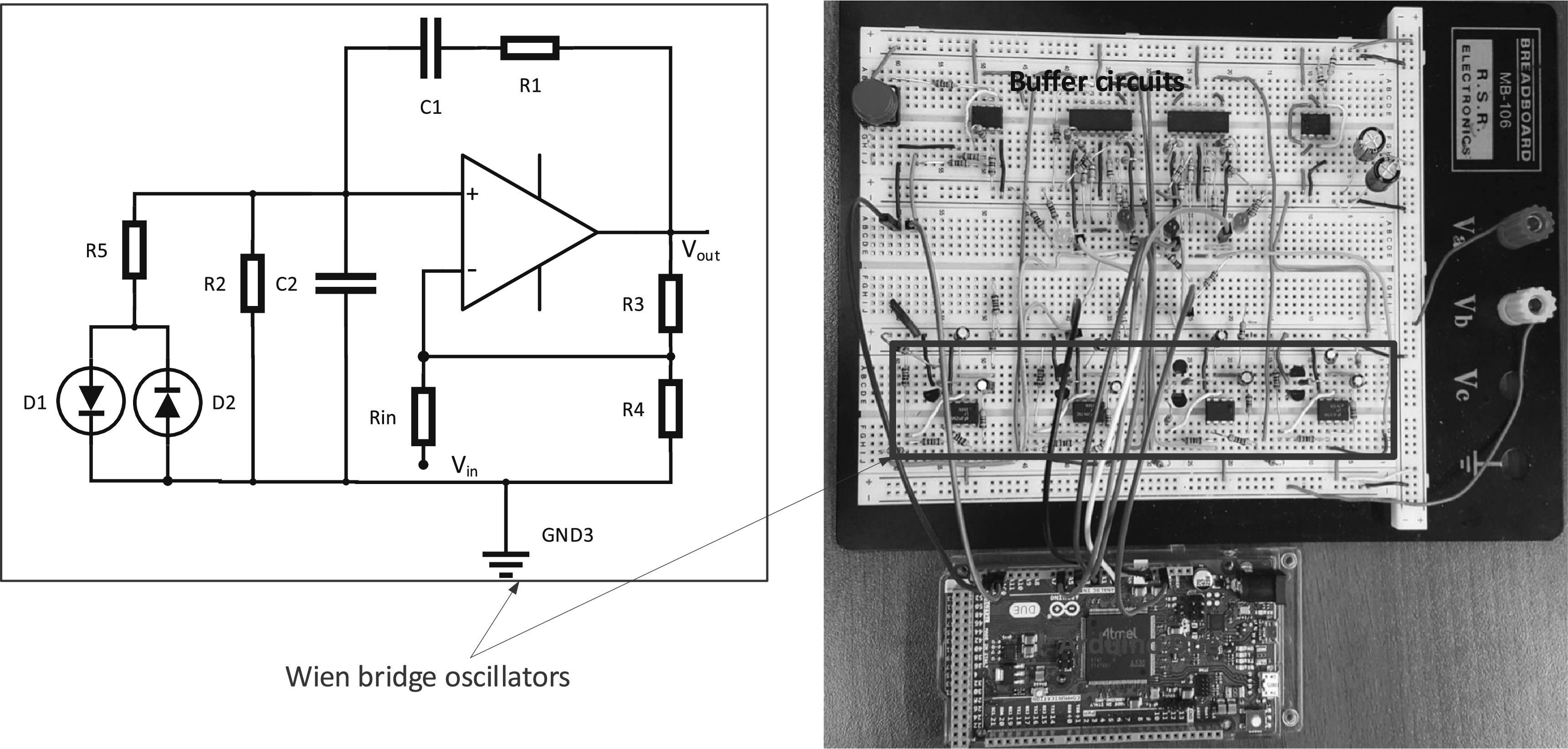
Experimental setup. The left panel shows the schematic of a Wien bridge oscillator. The image of the implemented circuit is shown in the right panel.

Given that the Arduino Due only measures positive voltages, buffers and offset circuitries were used to enable the measurement of the full waveform from each oscillator. As for the control input *u*(*t*), we used both analog outputs of the Arduino Due with a difference amplifier circuit to reproduce both positive and negative voltages. The control input was designed in Matlab and applied to the hardware using Matlab/Simulink, which was running the Arduino in real-time (figure 3).

The natural frequencies of four oscillators were: *ω*_1_ = 4.742 rad/s, *ω*_2_ = 4.776 rad/s, *ω*_3_ = 4.820 rad/s and *ω*_4_ = 4.91 rad/s, respectively. These oscillators were built using two LM741CN and two LF356N Op-Amps, a capacitor value C = 4.7*μ*F was used for all four oscillators (C1 = C2), transistors 2N3904 were used in diode mode as D1 and D2. The resistors R3 = 22 kΩ, R4 = 10 kΩ, R5 = 500 Ω and Rin = 100 kΩ were the same for all four oscillators. The resistors R1 = R2 were 56 kΩ, 53.9 kΩ, 48.8 kΩ, and 47 kΩ, for oscillators 1 to 4, respectively.

## 3. Results

The proposed iterative algorithm is a unified approach to controlling limit cycle oscillators, which offers flexibility in designing optimal controls for different objectives. For example, when tracking a specified reference trajectory is not required, our method can be used to design minimum energy controls to achieve a desired final state by setting the penalty matrix *Q* = 0. For such objectives, an arbitrary small terminal error, *φ*, can be achieved at the expense of input energy by tuning the matrices *R* and *F*, respectively. The flexibility to regulate the terminal error, tracking error, and applied input energy, by simply tuning the respective penalty matrices, enables our method for phase assignment [12], and control of spike timing [35, 36]. Furthermore, this method can be applied for designing real-time feedback controls by pre-computing the feedback gains and storing them in lookup tables as it is done in flight control [37].

### 3.1. Phase pattern formation in a network of HH oscillators

**Figure 4.**
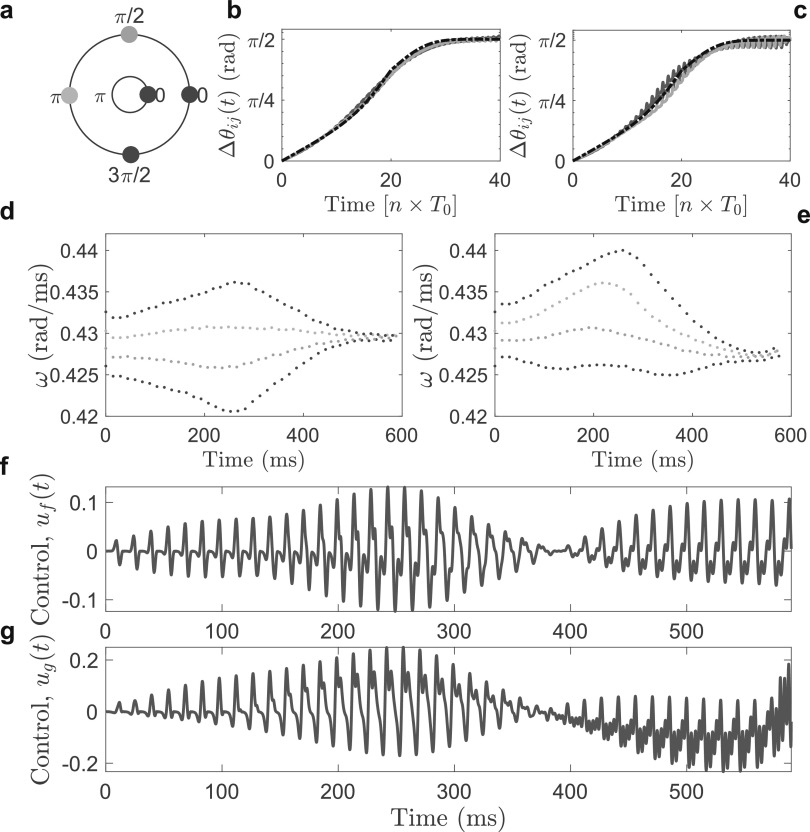
Phase assignment in a network of 4 Hodgkin-Huxley neurons by controlling the phase of each oscillator, and the phase differences between oscillators. Case-1: When the control input is designed to regulate the phase differences between oscillators. Case-2: When the control input is designed to follow the defined phase trajectory of each oscillator. (a) Initial and final phases (inner and outer circles, respectively) in both cases (b) Phase differences when the control algorithm is tracking a desired phase difference (black), in the time interval *t* ∈ [0, 40 × *T*_0_], where *T*_0_ = 14.75 ms (case-1). (c) Phase differences when tracking defined phase trajectories that give the desired phase pattern (case-2). (d)**-**(e) Neurons’ spiking frequencies during the control interval, corresponding to the trajectories in (b) and (c), respectively. Each colored dot represents one neuron. (f)-(g) Control signals that generated the trajectories in (b) and (c), respectively. The energy of the control *u*_*f*_ is *E* = 0.98 and the energy of *u*_*g*_ is *E* = 4.19. The natural frequencies are: (*ω*_1_, *ω*_2_, *ω*_3_, *ω*_4_) = (0.4207, 0.4264, 0.4322, 0.4379) rad/ms.

Control of the spatiotemporal patterns in neural networks using the minimum control energy is desired since the application of large inputs could be harmful, e.g. a strong electric field could harm neurons or some brain tissues. We illustrate the use of the proposed iterative tracking method by considering the problem of assigning a phase difference of Δ*θ*_ref_ = *π*/2 rad between successive neurons in an ensemble of four neurons. Figure 4(a) illustrates the final phase pattern at time *t*_*f*_ with a system of four HH neurons with natural frequencies (*ω*_1_, *ω*_2_, *ω*_3_, *ω*_4_) = (0.4207, 0.4264, 0.4322, 0.4379) rad/ms, and the neurons are ordered from the slowest (neuron 1, red circle in figure 4(a)) to the fastest (neuron 4, purple circle in figure 4(a)). We accomplish the displayed phase pattern, figure 4(a), by formulating the phase assignment problem as a tracking problem where each neuron tracks a predefined trajectory designed such that the desired phase pattern is achieved at the time *t*_*f*_ = 40 × *T*_0_ (*T*_0_ = 14.75 ms). A naive yet instinctive way to design the reference trajectories is to define a linear phase reference trajectory for the slowest neuron as *θ*_1,ref_(*t*) = *ω*_1_ *t*, with *t* ∈ [0, *t*_*f*_], and afterward define the reference trajectories of the subsequent neurons as *θ*_*j*,ref_(*t*) = *θ*_*j*−1,ref_(*t*) + Δ*θ*_ref_(*t*), with *j* = 2, 3, 4.

When tracking or assigning relative phase differences between heterogeneous oscillators, it is best to consider the system of phase differences, given by

\begin{eqnarray*}{\mathrm{\Delta }}{\dot{\theta }}_{j}={f}_{j}({\mathrm{\Theta }})-{f}_{j-1}({\mathrm{\Theta }})+({z}_{j}({\theta }_{j})-{z}_{j-1}({\theta }_{j-1}))u(t),\end{eqnarray*}

where Δ*θ*_*j*_ = *θ*_*j*_ − *θ*_*j*−1_, with *j* = 2, ⋯ ,*n*. Consequently, we define the vector of reference trajectories as ${\mathrm{\Delta }}{{\boldsymbol{\Theta }}}_{\mathrm{ref}}(t)=({\mathrm{\Delta }}{\theta }_{2,\mathrm{ref}},\cdots ,{\mathrm{\Delta }}{\theta }_{n,\mathrm{ref}})^{\prime} \in {{\mathbb{R}}}^{n-1}$, where Δ*θ*_*j*,ref_ is the reference trajectory of the phase difference between the *j* ^*th*^ and ${\left(j-1\right)}^{{th}}$ oscillators. This formulation results in a control with smaller energy and improved tracking accuracy. This is evident in figures 4(f) and (g), where the control *u*_*f*_, obtained by regulating only the phase differences, has a lower amplitude and 4.28 times less energy than the control *u*_*g*_ which regulates the trajectory of individual oscillators. Additionally, the resulting terminal error reduces to 5 × 10^−3^ from 16 × 10^−3^ when only phase differences are controlled. The penalty matrices corresponding to input cost, running cost, and terminal cost are *R* = 0.025, $Q(t)=\tfrac{0.004t}{{t}_{f}}I$, and *F* = 0.5*I*, where *I* is a *n* × *n* identity matrix with *n* denoting the system dimension, for both scenarios, i.e. whether regulating the phase of each oscillator (*n* = 4) or the phase differences between the oscillators (*n* = 3).

Figure 4(e) reveals that the frequency of the slowest oscillator (red dots) remains constant after applying the control *u*_*g*_, unlike the case when the control *u*_*f*_ is applied (figure 4(d)). The underlying reason is that we define a linear phase reference trajectory *θ*_1,ref_(*t*), i.e. constant frequency, for the slowest neuron while designing *u*_*g*_. No such constraints on the slowest oscillator’s phase trajectory are placed to design *u*_*f*_. In the first case, *u*_*g*_ control, the frequency of the first oscillator is kept constant, which causes the remaining oscillators to speed up more than the second case, *u*_*f*_ control, to get to the proper phase configuration in the allocated time, *t*_*f*_ = 590 ms.

It is a well-established fact that increasing the spiking frequency of HH neurons demands more control energy than decreasing it. This can also be observed by examining the PRCs of the HH neurons, which are asymmetric with respect to the *x*-axis (see figure 1(a) in Supplementary Materials), making it more difficult to increase the spiking rate. A similar response is observed for a single neuron where the control amplitude to reduce the spiking period from 14.6 ms to 13.8 ms is almost double of that to increase the period from 14.6 ms to 15.6 ms (figures 1(c) and (d), Supplementary Materials).

**Figure 5.**
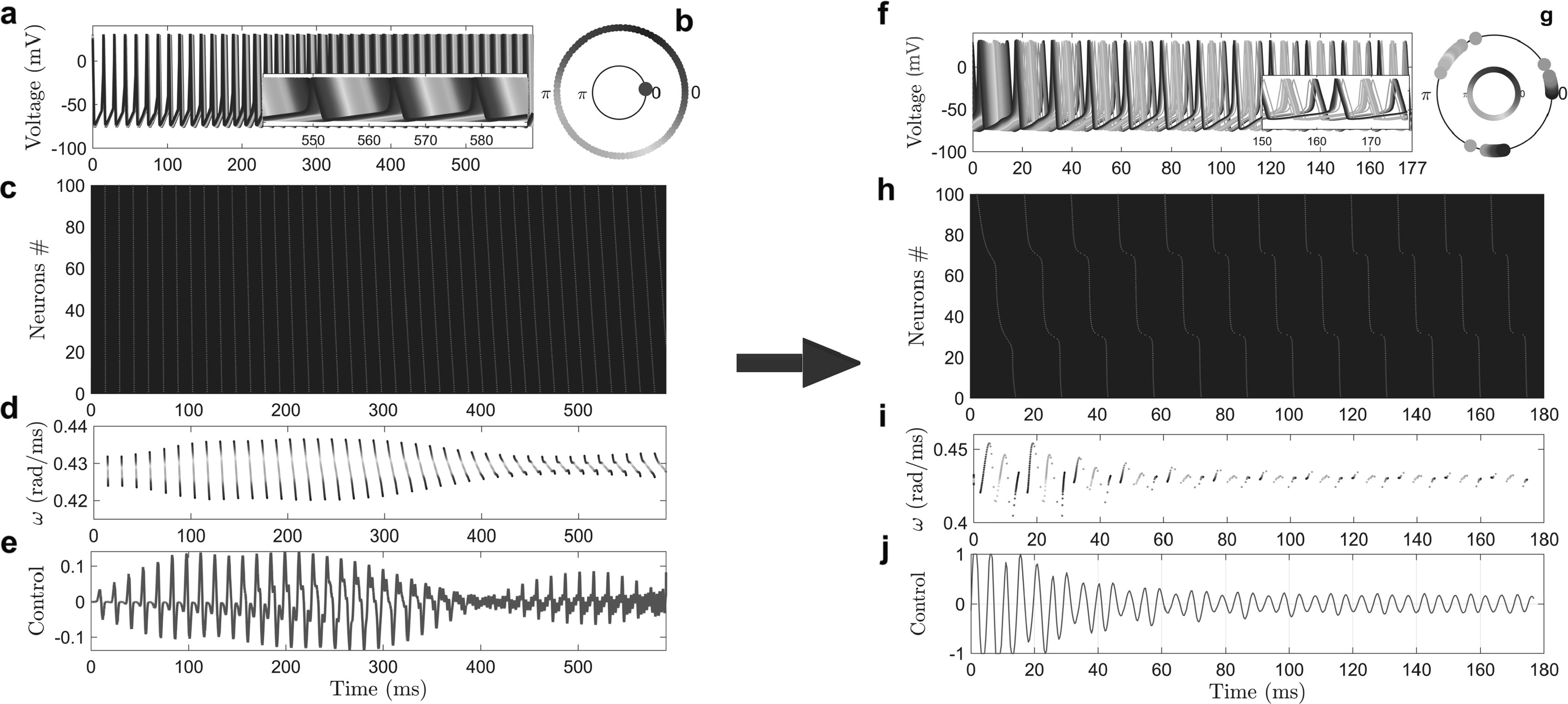
Uniform desynchronization and cluster formation in a population of 100 uncoupled HH neurons. (a), (f) Membrane voltages. (b), (g) Initial and final phase distributions of the membrane voltage phases. The inner circle shows the initial phases and the outer circle shows the final phases. (c), (h) Spiking patterns of the neurons. (d), (i) Instantaneous measured frequencies of each neuron during the control interval. Each colored dot represents one neuron. (e), (j) Control signal. The final phase pattern of the left panel (Outer circle of (b)) is taken as the initial phase distribution of the right panel (inner circle of (g)). The three clusters in the right panel contain neurons with frequencies in (*ω*_1*l*_, *ω*_1*h*_) = (0.42605, 0.42825) rad/ms, (*ω*_2*l*_, *ω*_2*h*_) = (0.42831, 0.43011) rad/ms and (*ω*_3*l*_, *ω*_3*h*_) = (0.43017, 0.43258) rad/ms, respectively.

In addition to the uniform phase assignment, we consider desynchronization and cluster-formation in a population of 100 HH neurons with uniformly distributed frequencies in [0.4261, 0.4326] rad/ms (see figure 5). Specifically, first, we design a control input to uniformly desynchronize the population from identical initial phases in time *t*_*f*_ = 40 × *T*_0_, where *T*_0_ = 14.75 ms. Once the population is desynchronized, we design another control input that drives the desynchronized ensemble into 3 uniformly distributed clusters in a predefined time 12 × *T*_0_. The control design is carried out with 12 HH neurons, and the resulting control input is then applied to 100 HH neurons, with the results shown in figure 5(g). Specifically, we select 12 HH neurons with 4 neurons taken from each frequency group, (*ω*_1*l*_, *ω*_1*h*_) = (0.42605, 0.42825) rad/ms, (*ω*_2*l*_, *ω*_2*h*_) = (0.42831, 0.43011) rad/ms and (*ω*_3*l*_, *ω*_3*h*_) = (0.43017, 0.43258) rad/ms, such that the boundaries of frequency groups are included in the selected neurons of each group. This is to ensure that the designed control input works as expected when applied to 100 HH neurons. The penalty matrices are taken as *R* = 1, $Q(t)=\tfrac{0.2t}{{t}_{f}}I$, and *F* = 2*I* for both uniform desynchronization and cluster formation. These numerical simulations illustrate that the proposed control strategy could generate complex patterns from arbitrary initial conditions in a large population of nonlinear oscillators.

### 3.2. Information processing and encoding in networks of oscillators

It is well-accepted that neuronal networks encode environmental information into firing patterns [38–42]. For instance, olfactory systems process scent cues by encoding them into complex spatiotemporal patterns [43], and brain functions, such as memory and information processing, are determined by sequential patterns [44, 45]. Therefore, control algorithms for the effective synthesis of stimuli that can create arbitrary spatiotemporal firing patterns will be crucial for neural coding and information processing. The prospect of controlling the firing sequence of neurons also has applications in the entertainment and medical industries. For example, being able to stimulate the neurons in the olfactory system to simulate scents in the era of 3D videography will enhance the entertainment experience, and the ability to activate the neurons responsible for transferring visual images to the brain will aid in the treatment of vision impairment [5, 46].

**Figure 6.**
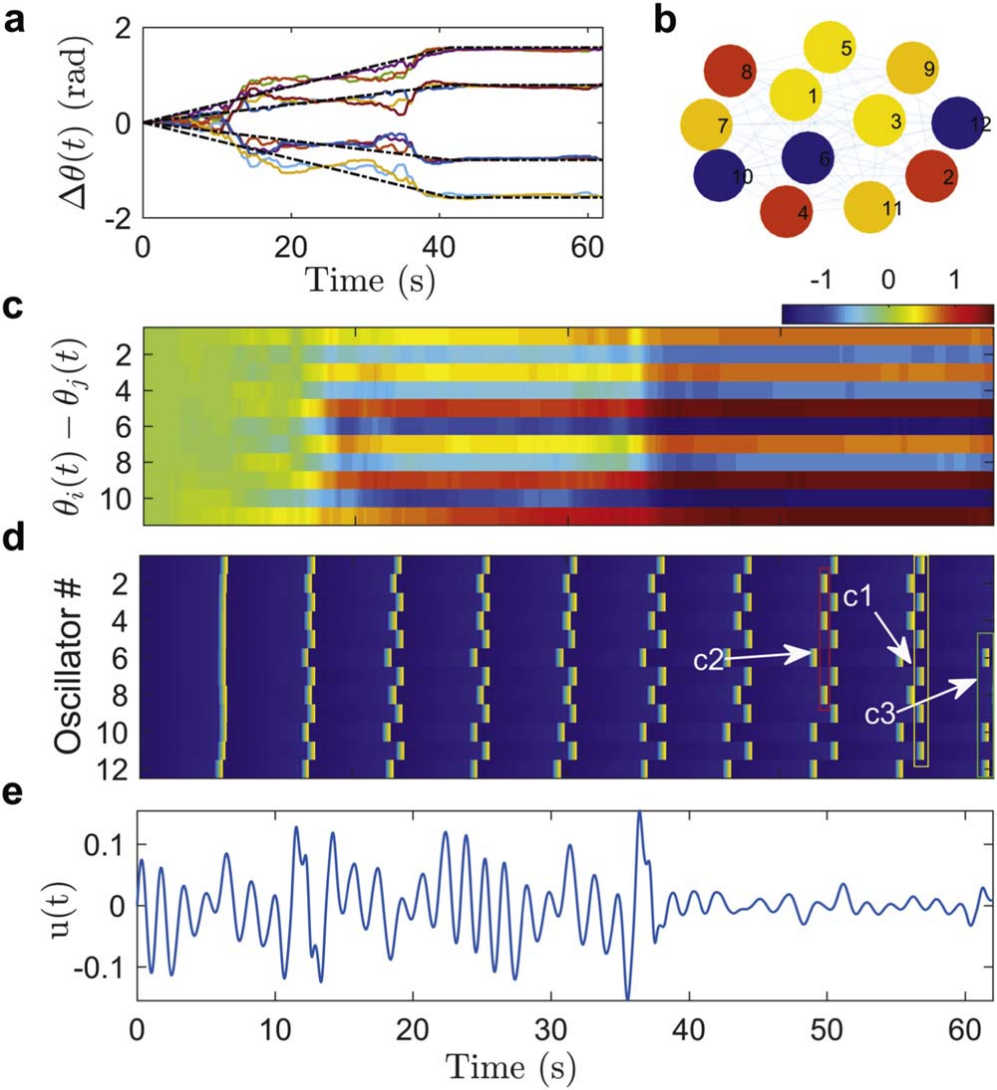
Information coding using phase assignment. (a) Phase difference trajectories. (b) Phase distribution of the twelve sinusoidal PRC oscillators on the network. (c) Encoding of information as a colored barcode representing relative phases between oscillators. (d) Encoding of information into firing patterns. The labels *c*_1_, *c*_2,_ and *c*_3_ delineate oscillators in the same cluster. (e) Broadcast control that generated the phase pattern. The *i* ^*th*^ oscillator in the network is simulated using sinusoidal coupling functions with the dynamics $\dot{{\theta }_{i}}={\omega }_{i}+\tfrac{\sigma }{12}{\sum }_{j=1}^{12}\sin ({\theta }_{j}-{\theta }_{i})+{a}_{i}\sin ({\theta }_{i})u(t)$. The parameters used are: *ω*_*i*_ ∈ [0.99, 1.01], *a*_*i*_ ∈ [1.82, 2], and coupling *σ* = 0.042.

Consider the all-to-all coupled network of 12 sinusoidal PRC neurons shown in figure 6(b), we design a control input that forms 3 different clusters such that neurons (1, 3, 5, 7, 9, 11) are in one cluster (c1), while the neurons (2, 4, 8) and (6, 10, 12) form the remaining 2 clusters (c2 and c3, respectively) and the neurons in c2 and c3 have a phase difference of −*π*/4 and *π*/2 with the neurons in c1. The designed control input and the phase difference trajectories, within successive neurons, are shown in figures 6(e) and (a). The penalty matrices are taken as *R* = 4, $Q(t)=\tfrac{0.08t}{{t}_{f}}I$, and *F* = 10*I* where *t*_*f*_ = 61.8 s. Using this synchronization structure, we portray two different schemes that could be utilized to, hypothetically, encode information into the spiking patterns of a neuronal network using the relative phase differences (figure 6(c)) or the phase of firing (figure 6(d)). Firstly, the encoding depicted in figure 6(c), which resembles a color barcode, can be utilized to encode information as various color patterns according to the relative phases of the oscillators. Secondly, the information can also be encoded into the phase of the firing of each neuron, as shown in figure 6(d). These schemes reveal that the encoding or storage capacity of oscillatory networks remains significant, even with a small number of neurons.

**Figure 7.**
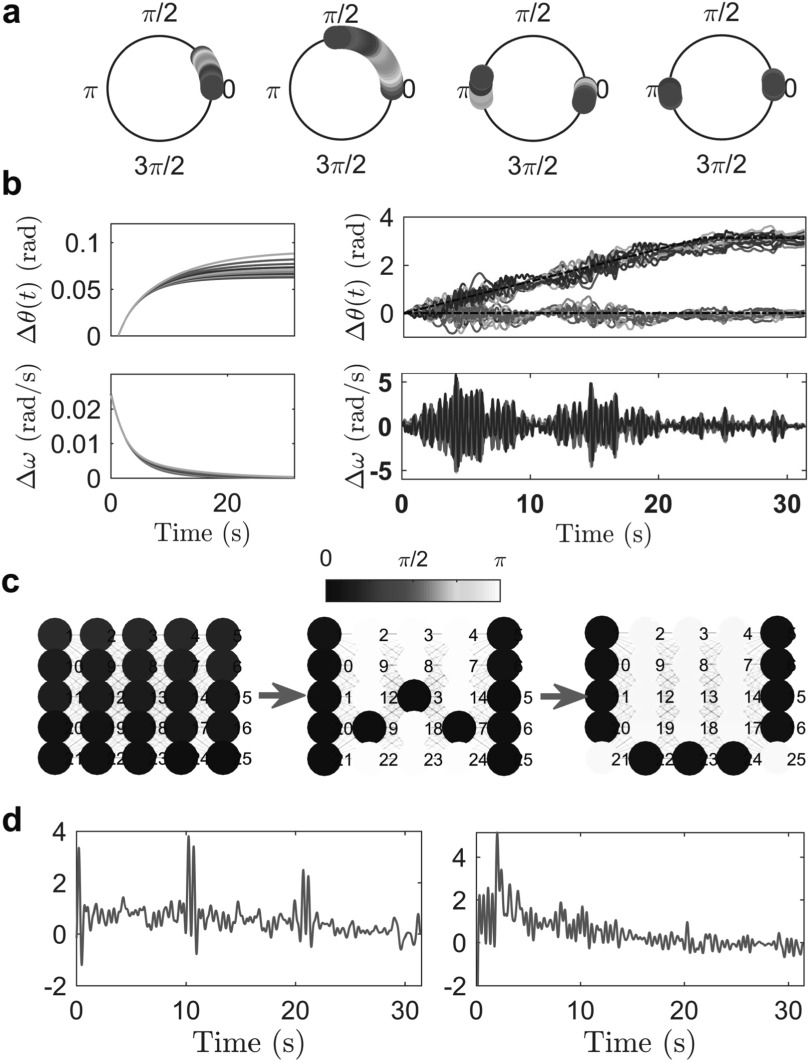
Pattern formation in a globally coupled network of SNIPER PRC oscillators. (a) Oscillator’s phase distributions on the unit circle. From left to right: initial phases, final phases for the uncontrolled network, final phases of the pattern ‘W’, and the final phases of the pattern ‘U’. (b) Trajectories of the phase differences (top panels) and frequency differences (bottom panels). Left panel: free evolution, right panel: controlled trajectories. (c) Formation of letter patterns on a 5 × 5 grid of oscillators. Starting with oscillators phases, *θ*_*i*_ ∈ [0, *π*/4] rad, the pattern of ‘W’ was formed, followed by ‘U’, with ‘W’ as an initial condition. (d) Control signals. The patterns ‘W’ and ‘U’ were obtained by successively applying the inputs in the left and right panels, respectively. The *i* ^*th*^ oscillator, *i* = 1,…,*n*, is described by the dynamics $\dot{{\theta }_{i}}={\omega }_{i}+\tfrac{\sigma }{n}{\sum }_{j=1}^{n}\sin ({\theta }_{j}-{\theta }_{i})+{a}_{i}(1-\cos {\theta }_{i})u(t)$. The parameters of the network are *ω*_*i*_ ∈ [0.8, 1.2], *a*_*i*_ ∈ [1, 2], and the coupling *σ* = 0.3.

Now, we show that our control framework can be directly used to encode information by producing spatial patterns in a globally coupled network of 25 heterogeneous SNIPER oscillators, ${z}_{i}({\theta }_{i})={a}_{i}(1-\cos ({\theta }_{i}))$ where *a*_*i*_ is a model-dependent constant [32]. SNIPER oscillators describe neurons near a SNIPER bifurcation (i.e. a saddle-node bifurcation on a periodic orbit) [36]. Figure 7 displays the pattern formation from a uniform initial phase distribution to the pattern ‘W’ (figure 7(c), middle panel) and then switch to the ‘U’ pattern (figure 7(c), right panel) using the derived control sequence depicted in figure 7(d) (the two letters W and U stand for Washington University). These letter patterns are obtained by assigning the same phase, e.g. 0rad, to all the oscillators forming the letter pattern, while assigning the remaining oscillators a phase equal to *π* rad. The trajectories of the phase differences in the top right panel of figure 7(b) show how the *π* phase difference between oscillators is assigned following a linear reference trajectory for 23 seconds. Once the oscillators are controlled to the right configuration, the last 8 seconds of the control signals continued to maintain the phase differences for both patterns, respectively. The oscillator phases on a unit circle are displayed in figure 7(a), where the first panel depicts the initial oscillator phases and the second, third, and fourth panels display the uncontrolled final phases, and the final phases corresponding to pattern 'W’ and ‘U.’ The penalty matrices are *R* = 0.25, $Q(t)=\tfrac{0.05t}{{t}_{f}}I$, and *F* = 3*I*, where *t*_*f*_ = 31 s.

Our developed broadcast control strategy is particularly important for applications involving manipulating ensemble systems with limited control sources. For example, the number of electrodes needed can be significantly reduced for retinal implants to transmit visual information to the visual cortex. In particular, using one electrode may be sufficient to control all the neurons in a given surface area. Such capability is quantified by the controllability property, which we will elaborate on in the next section.

### 3.3. Controllability and information encoding

The tasks depicted in figure 6 and figure 7 consisted of designing a control capable of steering the population of oscillators from an initial state (pattern) ***x***_0_ to a desired final state (pattern) ***x***_1_. This was possible because ***x***_1_ was reachable from ***x***_0_ by the application of a control input, as evident by the numerical simulations in these figures. The collection of points that can be reached from ***x***_0_ forms the reachable set, denoted ${{ \mathcal R }}_{{{\boldsymbol{x}}}_{{\bf{0}}}}$. Consider a nonlinear system of the form,

\begin{eqnarray*}\dot{{\boldsymbol{x}}}(t)={\boldsymbol{f}}({\boldsymbol{x}}(t))+u(t){\boldsymbol{g}}({\boldsymbol{x}}(t)),\ {\boldsymbol{x}}(0)={{\boldsymbol{x}}}_{0},\end{eqnarray*}

where ***f*** and ***g*** are smooth vector fields on a manifold $M\subset {{\mathbb{R}}}^{n}$, and the solution of $\dot{{\boldsymbol{x}}}(t)={\boldsymbol{f}}({\boldsymbol{x}}(t))$ is periodic for each ***x***_0_ ∈ *M*. Then, the reachable set ${{ \mathcal R }}_{{{\boldsymbol{x}}}_{{\bf{0}}}}$ is characterized by the exponential map, ${\{\exp \{{\boldsymbol{f}},{\boldsymbol{g}}\}{}_{{LA}}\}}_{G}{{\boldsymbol{x}}}_{0}$, of the Lie algebra generated by ***f*** and ***g***, denoted {***f***, ***g***}_*LA*_, which can be determined by computing recursive Lie brackets [47]. The Lie algebra {***f***, ***g***}_*LA*_ generated by ***f*** and ***g*** can be determined by computing the recursive Lie brackets ${{ad}}_{{\boldsymbol{f}}}^{k}{\boldsymbol{g}}({\boldsymbol{x}})=[{\boldsymbol{f}},{{ad}}_{{\boldsymbol{f}}}^{k-1}{\boldsymbol{g}}]({\boldsymbol{x}})$ of ***f*** and ***g***, where ${{ad}}_{{\boldsymbol{f}}}^{0}{\boldsymbol{g}}({\boldsymbol{x}})={\boldsymbol{g}}({\boldsymbol{x}})$ and ${{ad}}_{{\boldsymbol{f}}}^{1}{\boldsymbol{g}}({\boldsymbol{x}})=[{\boldsymbol{f}},{\boldsymbol{g}}]({\boldsymbol{x}})=\tfrac{\partial {\boldsymbol{g}}}{\partial {\boldsymbol{x}}}{\boldsymbol{f}}-\tfrac{\partial {\boldsymbol{f}}}{\partial {\boldsymbol{x}}}{\boldsymbol{g}}$. It follows that if {***f***, ***g***}_*LA*_ spans ${{\mathbb{R}}}^{n}$ at all points ***x*** ∈ *M*, then any state in *M* can be reached from any initial condition; hence the system in equation (10) is controllable [48]. Ample discussions on the reachability and controllability of nonlinear systems can be found in [47, 49].

The information encoding capacity of a neuronal network, using the schemes in figures 6 and 7, depends on the number of heterogeneous neurons and the controllability property of the network. If the neuronal system is controllable, any spiking pattern can be generated, which in turn determines its information encoding capacity. Here, we derive the conditions under which an ensemble of oscillators with constant baseline dynamics, i.e. *f*_*i*_(*θ*) = *ω*_*i*_, is controllable. Because PRCs are periodic functions, without loss of generality, we may focus on analyzing oscillators with PRCs expressed by truncated Fourier-series. Specifically, we consider the commonly-used phase model describing a population of oscillators with ${\boldsymbol{f}}({\mathrm{\Theta }})={\left({\omega }_{1},\ldots ,{\omega }_{n}\right)}^{{\prime} }$ and ${\boldsymbol{Z}}({\mathrm{\Theta }})={\left({a}_{1}z({\theta }_{1}),\ldots ,{a}_{n}z({\theta }_{n})\right)}^{{\prime} }$, where *a*_*i*_ > 0, *i* = 1,…,*n*, are model dependent parameters, and *z*(*θ*_*i*_) is the PRC of the oscillator *i*. We show that such an oscillator ensemble is controllable if the oscillators have distinct natural frequencies, i.e. *ω*_*i*_ ≠ *ω*_*j*_ for *i* ≠ *j* (see Supplementary Materials for proof). Note that this result is a generalization of the result established in [48], where only special forms of PRCs, e.g. SNIPER PRC, $z({\theta }_{i})=(1-\cos ({\theta }_{i}))$, or sinusoidal PRC, $z({\theta }_{i})=\sin ({\theta }_{i})$, were discussed.

### 3.4. Partial controllability and degradation of information encoding

When a neuronal ensemble is partially controllable, only limited phase patterns can be generated with control inputs. This in turn reduces the capacity of information encoding and impairs information processing in the network. This phenomenon can be simply illuminated with a chain network of three Kuramoto oscillators, modeled by

\begin{eqnarray*}\begin{array}{rcl}\dot{{\theta }_{1}} &amp; = &amp; {\omega }_{1}+\sigma [\sin ({\theta }_{2}-{\theta }_{1})]+{z}_{1}u(t),\\ \dot{{\theta }_{2}} &amp; = &amp; {\omega }_{2}+\sigma [\sin ({\theta }_{1}-{\theta }_{2})\\ &amp; &amp; +\sin ({\theta }_{3}-{\theta }_{2})]+{z}_{2}u(t),\\ \dot{{\theta }_{3}} &amp; = &amp; {\omega }_{3}+\sigma [\sin ({\theta }_{2}-{\theta }_{3})]+{z}_{3}u(t),\end{array}\end{eqnarray*}

evolving on the state-space manifold $M={{\mathbb{T}}}^{3}$, a 3-Torus, where *σ* is the coupling strength and *u*(*t*) is the common control input. Controllability of this network is characterized by the Lie algebra generated by the vector fields ***f*** and ***Z***, where ***f*** = $({\omega }_{1}+\sigma [\sin ({\theta }_{2}-{\theta }_{1})]$, ${\omega }_{2}+\sigma [\sin ({\theta }_{1}-{\theta }_{2})+\sin ({\theta }_{3}-{\theta }_{2})]$, ${\omega }_{3}+\sigma [\sin ({\theta }_{2}-{\theta }_{3})])^{\prime} $ and ***Z*** = $({z}_{1},{z}_{2},{z}_{3})^{\prime} $. Note that if the input gains, *z*_1_, *z*_2_, and *z*_3_, are equal, and the natural frequencies, *ω*_1_, *ω*_2_, and *ω*_3_, are identical, then this network is not controllable. This can be shown by evaluating the recursive Lie brackets ${{ad}}_{{\boldsymbol{f}}}^{k}{\boldsymbol{Z}}$, that are ${{ad}}_{{\boldsymbol{f}}}^{0}{\boldsymbol{Z}}={\left({z}_{1},{z}_{1},{z}_{1}\right)}^{{\prime} }$ and ${{ad}}_{{\boldsymbol{f}}}^{k}{\boldsymbol{Z}}={\bf{0}}$, $k\in {{\mathbb{Z}}}^{+}$. Thus, $\{{\boldsymbol{f}},{{ad}}_{{\boldsymbol{f}}}^{k}{\boldsymbol{Z}}\},k\in {{\mathbb{Z}}}^{+}$ does not span ${{\mathbb{R}}}^{3}$ at all ${\boldsymbol{\Theta }}\in {{\mathbb{T}}}^{3}$, which means the system is not controllable. In general, other than this special case, there are instances where this network is partially controllable over a submanifold of *M*. For example, when (*ω*_1_, *ω*_2_, *ω*_3_) = (1, 1.1, 1.2) and ${\boldsymbol{Z}}=(0.8,1.0,1.2)^{\prime} $, the network is partially controllable on the submanifold ${ \mathcal S }=\{{\boldsymbol{\Theta }}=({\theta }_{1},{\theta }_{2},{\theta }_{3})^{\prime} :({\theta }_{2}-{\theta }_{1})=({\theta }_{3}-{\theta }_{2})\}$ as {***f***, ***Z***}_*LA*_ only spans ${{\mathbb{R}}}^{2}$, and this implies that arbitrary phase patterns are not achievable if starting with the initial phase ${{\mathrm{\Theta }}}_{0}\in { \mathcal S }$ (see Supplementary Materials for more details).

As the uncontrollability of a network leads to the reduction in the capability of forming diverse phase patterns, this may be compensated by using networks of greater size, composed of more oscillators, to enhance encoding capacity. This is analogous to a digital system that encodes information as 0's and 1's, requiring a sufficient number of bits to represent data. Similarly, the loss of controllability in the brain circuitry could lead to the loss of brain functions due to the inability of the neural network to reproduce the precise spiking pattern that contains the necessary information.

### 3.5. Real time control of Wien bridge oscillators

**Figure 8.**
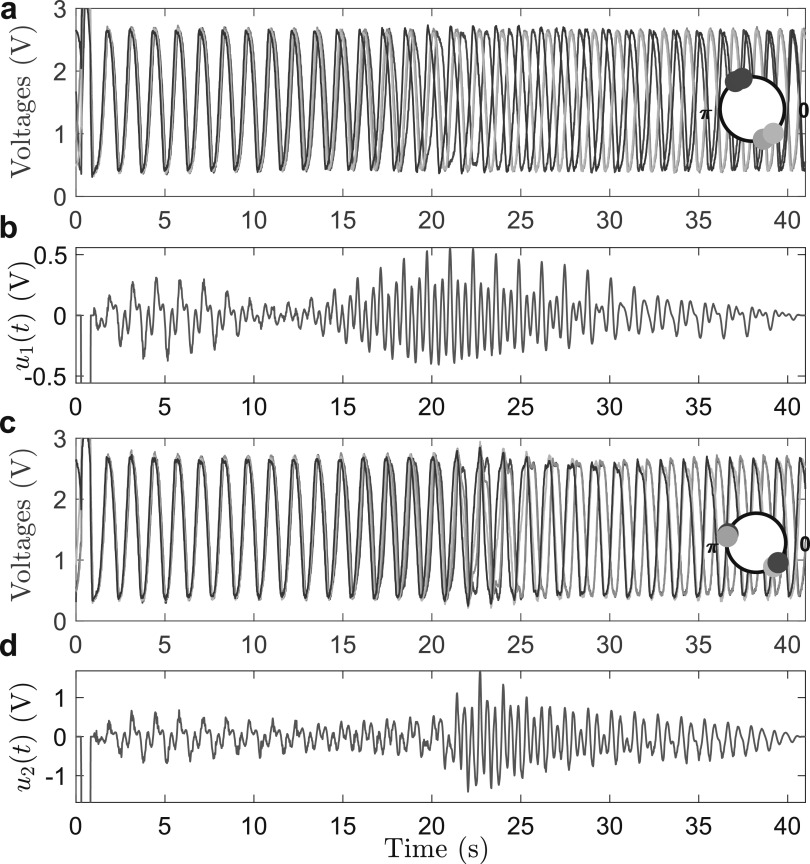
Two-cluster formation with four Wien bridge oscillators. (a) Output voltages of the oscillators during the formation of the clusters {1, 4} and {2, 3}. The inset shows the final phases of the oscillator ensemble. (b) Control input *u*_1_(*t*) that creates the clusters in (a). (c) Output voltages of the oscillators during the formation of the clusters {1, 2} and {3, 4}. (d) Control input *u*_2_(*t*) that creates the clusters in (c). The four oscillators have natural frequencies (*ω*_1_, *ω*_2_, *ω*_3_, *ω*_4_) = (4.742, 4.776, 4.820, 4.91) rad/s.

We apply the developed iterative algorithm to four heterogeneous Wien bridge oscillators to form two clusters, each consisting of two oscillators. For convenience, we refer to the slowest and the fastest oscillator as oscillators 1 and 4, respectively. We perform two different phase assignment tasks: For the first task, we aim to form the clusters {1, 4} and {2, 3} with *π* rad apart (figure 8(a)). For the second task, we form the clusters {1, 2} and {3, 4} with *π* rad apart (figure 8(c)).

The reference trajectory in these two examples had three parts: (1) a constant section that maintained the oscillators synchronized for 10 sec, (2) a ramp section that placed the oscillators in the right phase configuration (clusters), and (3) another constant section to maintain the clusters. Additionally, we apply a 5-volt resetting pulse at the start of the experiment to ensure that each oscillator has identical phases since we assume similar initial phases while estimating the control input.

Note, however, that designing the control using the phase difference equation (9) only requires the knowledge of the initial phase differences between the oscillators. In contrast, if the control design is done using equation (3), one needs to know the initial phases of the oscillators to design the control input. In the case of unknown initial phases, applying any synchronizing control or a resetting pulse will suffice.

## 4. Discussion

In this paper, we develop a unified iterative control framework for controlling spatiotemporal patterns in an ensemble of limit-cycle oscillators. We illustrate that the optimal tracking control can be precisely computed by knowing the phase dynamics of the system. We show the versatility and efficacy of the control algorithm through various numerical simulations and experiments. Further, we characterize the controllability of neuronal ensembles using tools from geometric control theory and show its implications on the information encoding capacity in neural networks.

One of the main ideas of our work is to transform the phase assignment problem into a tracking problem. By recasting the phase assignment problem as a tracking problem, we gain control over the phase trajectories at each moment and the time required to attain the desired phase pattern, which is useful for rapid cardiac resynchronization and fast jet lag recovery. Furthermore, unlike the existing methods for phase assignment [12, 45], which require sufficient non-linearity of the PRC to realize the desired phase pattern, our method merely requires reachability from the initial to the target phase configuration.

The control algorithm presented here has the potential to make an impact on a wide range of applications, ranging from the treatment of neurological pathologies such as Parkinson’s disease and epilepsy [50, 51] to the design of neurocomputers [52, 53]. The applicability of our open-loop control methodology to various applications can be attributed to two factors: first, the robustness of our methods, as proved by experimental results, and second, the fact that feedback information of individual units is not required to synthesize a control rule. The presented broadcast control strategy will also have a significant impact on clinical applications, such as retinal implants, by reducing the number of required electrodes for transmitting visual information to the visual cortex via optic neurons. Although the core of this work considered neuron oscillator phase models, the methods and analysis presented here are applicable to any limit-cycle oscillators, e.g. pulsating cardiac cells generating heartbeats, which are often modeled as reaction-diffusion systems [33]. Furthermore, due to the algorithm’s iterative nature, our suggested control method is computationally tractable, making it suitable for large networks. In each iteration, the computation cost is just ${ \mathcal O }({n}^{2}M)$ to solve three ordinary differential equations, where *n* is the system dimension and *M* is the number of time steps. All the numerical experiments are implemented in Matlab on a single workstation with Xeon Gold 6144 3.5 GHz processor and 192 GB memory, and the simulation time is within 2-3 H for all the examples.

It is also essential to acknowledge and discuss some limitations of our study. First, we design the control waveform based on phase models and then apply the designed control input to the original high-dimensional system. Suppose the designed control input is not weak. In that case, the phase models will not accurately reflect the actual phase dynamics of the original system, and the output obtained from applying the control input to the original system will not be as desired. However, by properly regulating the input and state penalty matrices, sufficiently weak input can be obtained to ensure the validity of phase models at the cost of small phase assignment errors. Second, while doing controllability analysis, we neglect the connection between oscillator units. Even though our results could be applied to weakly coupled oscillator ensembles, a detailed investigation of the influence of coupling on the network’s information encoding capability, i.e. controllability property, is still required, which forms the foundation for future works.

## Data Availability

All data that support the findings of this study are included within the article (and any supplementary files).

## Author contributions

J -S L designed the research; W B, B S, and J -S L performed the research; W B performed numerical simulations; W B and B S prepared figures; and W B, B S, and J -S L wrote the paper and Supplementary Materials.

## Competing interests

The authors declare no competing interests.
